# Investigating the Occurrence of Soil-Transmitted Parasites Contaminating Soil, Vegetables, and Green Fodder in the East of Nile Delta, Egypt

**DOI:** 10.1155/2023/6300563

**Published:** 2023-06-06

**Authors:** Samah H. Yahia, Samia E. Etewa, Abd Allah A. Al Hoot, Salwa Z. Arafa, Nesreen S. Saleh, Mohamed H. Sarhan, Suzan I. Rashad, Shimaa S. Hassan

**Affiliations:** ^1^Medical Parasitology Department, Faculty of Medicine, Zagazig University, Zagazig, Egypt; ^2^Zoology Department, Faculty of Science, Zagazig University, Zagazig, Egypt; ^3^Zoology Department, Faculty of Arts and Sciences Al-Wahat, Benghazi University, Benghazi, Libya; ^4^Water and Soil Pollutanta Laboratory, Regional Research Center in Sharkia Governorate, Academy of Scientific Research and Technology, Egypt

## Abstract

**Background:**

Food-borne parasites are major sources of human and animal illness, posing severe health risks in places with contaminated soil, poor water quality, cleanliness, and poor sanitation. The usage of untreated organic fertilizers arising from the excreta of the parasites' definitive hosts either man or animal pollutes the agricultural soil and is reflected in its products of vegetables and green fodders causing serious health problems. Therefore, to the best of our knowledge, this will be the first study that investigated the combination of parasitic contamination of the agricultural soil and its products of raw eaten vegetables and green fodder in East Nile Delta, Egypt.

**Aim:**

The purpose of this study was to investigate the type and degree of contamination caused by parasites in regularly used raw vegetables, green fodder, and soil samples collected from open fields in Egypt's East Nile Delta. *Study Procedures*. A cross-sectional study comprised a simple random collection of 400 soil samples, 180 green fodder samples, and as well as 400 vegetable samples, including lettuce, radish, coriander, parsley, dill, watercress, tomatoes, green pepper, cucumber, and carrot, that were gathered throughout one year period from January to December 2021 to represent all seasons (winter, spring, summer, and autumn). The research locations were chosen from various open green fields and farming regions in Egypt's East Nile Delta producing ready-to-eat vegetables for human consumptions and planting green fodder for animal feeding. Concentrations, including sedimentation, and flotation, and staining techniques were used to recover the greatest number of parasitic life forms. The parasitic structures discovered were identified using biometric and imaging data and compared with known parasite morphology. Statistical analysis was performed with the SPSS software version 22 (IBM, Chicago, IL, USA). Data were presented in numbers and percentages. *P*-values equal to or less than 0.05 were considered to be statistically significant. The difference in parasitic contamination among the different categories was compared using the chi-square test.

**Results:**

In this investigation, 243 out of 400 soil samples (60.7%) confirmed positive for parasitic contamination (*P* < 0.05). Various parasitic life forms were significantly found in 249 out of 400 (62.25%) of the vegetable samples, with (65.1%) of them harboring one parasite species, whereas 9.2% significantly contained up to three parasites. *Ascaris* eggs, *Trichuris* eggs, and *Giardia* cysts were the most prevalent parasites, which were predominantly isolated from vegetables with uneven surfaces. 109 of 180 (60.0%) green fodder samples confirmed insignificantly positive for parasitic pollution. The proportion of parasite contamination in vegetable samples was insignificant although the highest was in spring (29.3%), followed by summer (27.7%), whereas it is significant in autumn (24.5%). The prevalence rate was the lowest in winter (20.1%). *Conclusion and Recommendations*. Our findings demonstrated a significant load of parasites notably the soil-transmitted parasitic infection in raw vegetables and green fodder cultivated in open fields as well as in their mother soil in the east of the Nile Delta, Egypt. These results confirm the urgent need to deploy strict control measures to the soil, especially during the pre-harvest period of raw eaten vegetables and green fodder, a critical step in reducing food-borne transmission of soil-transmitted parasites to man and animals.

## 1. Introduction

Parasitic helminths and protozoa are vastly diverse eukaryotic organisms that can infect humans and animals through a variety of transmission channels, including soil and food [1]. Food-borne pathogens, such as parasites, bacteria, and viruses, have been linked to a considerable number of human fatalities and illnesses [2], with parasites being more prevalent in fresh food than other types due to their greater capacity to remain in the environment [3]. Intestinal parasites, including soil transmitted helminths (STHs), are among the world's food-borne public health hazards, particularly in tropical and subtropical nations [4]. These infections share soil- and food-borne transmissions as major variables in their human population predominance because they discharge their eggs/larvae in the feces of infected hosts, contaminating food or polluting soil due to unhealthy hygienic practices [5, 6]. In an evaluation of the global relevance of the top 24 food-borne parasites weighted and based mostly on public health issues, *Ascaris lumbricoides* and *Trichuris trichiura* in fresh produce were rated 9th and 15th, respectively [7]. The concept that these parasites are confined to specific geographic locations because they are adapted to certain definitive hosts, selected intermediate hosts, and specific environmental circumstances is gradually being broken down due to factors related to climatic changes, international travel, and global food transport activities. Food-borne parasitic infections have been proliferated due to reasons, such as the globalization of dietary commerce, differences in food preferences, and consumption patterns [8]. Despite the tremendous breakthroughs in developing a specific and sensitive technique for detecting such parasites in human blood and stool, the identification of parasites eggs in environmental samples like vegetables and the soil, which is equally crucial for management, remains a difficult issue [9]. This may be due to the diverse nature of food-borne parasites, their low quantity in foodstuff, and their resistance to in vitro growth as well as the variety of food vehicles by which they may be transferred to consumers. Therefore, it is not surprising that the international system of food safety lacks a uniform validation mechanism for parasite-free food [10]. Food-borne parasites have not always received the attention they deserve; this is due to the lack of acute manifestations that delays treatment and assists in infection transmission [10].

Green vegetables are crucial elements in a balanced healthy human diet as they are cholesterol free and abundant with vitamins, dietary fiber, and minerals [11] that have been linked to lower risks of heart disease, stroke, and cancer [12]. The global interest in ingesting raw veggies has developed dramatically in recent decades as a result of changing eating habits of consumers seeking a healthy lifestyle of low thermal calories by eating fresh fruits and vegetables to keep the natural flavor and preserve their heat-labile nutrients [13]. In the era of globalization, where diseases transmitted by food cross all geographical, political, and educational boundaries, food-borne parasites may be spread and find new ways of establishment in multiple niches, exposing more human and animal hosts to infection [14]. Following the broad findings of many literature publications identifying vegetables as key carriers of parasitic diseases transmission, the fresh produce business has begun to pay attention to vegetables as a source of parasitic illnesses [15–17] as they can get contaminated at any point along the food manufacturing chain from when they are on the ground till the consumers' tables. Polluted soil or water, for example, might contribute to contamination before harvest. Different insect vectors and animal species used in the farming process, as well as human handling during processing and packaging or even at home, might all have a role in the parasitic contamination of green vegetables [18]. The surface morphology of various green vegetables, particularly leafy ones, promotes the attachment of sticky-natured parasite eggs and cysts that easily adhere to the matrix of these foods [19]. As a result, these food items have become crucial carriers for the spread of food-borne infections, with a rise in the number of outbreaks of food-borne illnesses related to fresh produce consumption [20].

Soil is an important vehicle that poses a significant role in the spread of intestinal parasites to humans and animal hosts. This matrix may get polluted as a result of the harmful practice of dumping fecal matter of infected hosts straight into the soil, which serves as an essential source of parasitic contamination of growing vegetables, or directly onto human hands [21]. Although it is well known that parasites do not multiply outside a living host, the regular consumption of ecologically grown products due to perceptions, such as organic farming, being more eco-friendly and free from chemicals, can artificially amplify the number of parasites contaminating vegetables leading to heavy infection in definitive hosts [22]. According to Food and Agriculture Organization (FAO), climatic extremes are one of the key drivers of food security and nutrition trends in developing nations [23]. These changes, such as excessive rains and floods, increase polluted water flow to agricultural land, amplifying its parasite load [24]. The practice of irrigation with untreated wastewater has extended in resource-constrained countries, which is often the case in the Middle East and North Africa as it boosts output while lowering production expenses by conserving fertilizer [25]. Contaminated soil has been linked to the parasitic contamination of vegetables in East and West African countries [18]. Due to the scarcity of water suitable for irrigation, as well as the increase in human population, the area of cultivated land for vegetables has increased significantly, necessitating the use of untreated irrigation water and the application of atypical organic manure for cultivation [26]. To lower the risk of STHs and other parasitic helminth infections to below the World Health Organization (WHO) recommendations, wastewater for agriculture should contain ≤1 helminth egg per liter. This necessitates sensitive detection and reliable quantification of parasitic contaminants in a variety of sample matrices, such as soil, irrigation water, and vegetables [27].

Given the increasing globalization of food supply chains and the high frequency and severity of parasite outbreaks in food, global surveillance of diverse parasite species in fresh vegetables and the soil is a public health imperative to ensure the safety of the foods we consume [28]. Therefore, the purpose of this study was to determine the extent of contamination of green ready-to-eat vegetables, green fodder, and soil from cultivated fields by soil-transmitted parasites using morphological characteristics visualized under a microscope. This was done not just to document its presence but also to aid in the development of preventive and control strategies, as well as to emphasize the importance of acknowledging its involvement in international food validation procedures.

## 2. Materials and Methods

This cross-sectional study was carried out between January and December 2021 to determine the level of parasitic contamination of soil, raw eaten vegetables, and green fodder by soil-transmitted parasites. The research locations were chosen from various open green fields and farming regions in Egypt's East Nile Delta producing ready-to-eat vegetables for human consumption and planting green fodder for animal feeding; these areas are known for producing and supplying large quantities of fresh products to all areas in Egypt. During the study period, the accessible and collected samples were 400 soil samples, 400 raw-eaten vegetables, and 180 green fodder samples.

### 2.1. Ethical Statement

The Ethics Committee of the Faculty of Medicine at Zagazig University in Egypt granted ethical permission for this study (ZU-IRB#:10067/22-11-2022). The farmers were not having any kind of conversation. The proprietors of the fields signed written permission to gather samples.

### 2.2. Data Quality Assurance

Training was given to sample collectors about where, when, and how to choose, collect, and transport the samples. The quality of reagents and instruments was checked by each laboratory principal. The samples were also checked for serial number, weight, and methods of collection.

For quality assurance, 10% of the samples were randomly selected and re-examined by an experienced laboratory technologist. The completeness of the required data was regularly checked by the supervisors. Sample collection, transportation, and accurate results registration were done according to the operational procedure standards.

### 2.3. Sample Collection Procedures

#### 2.3.1. Vegetables and Green Fodder Samples

**Figure 1:**
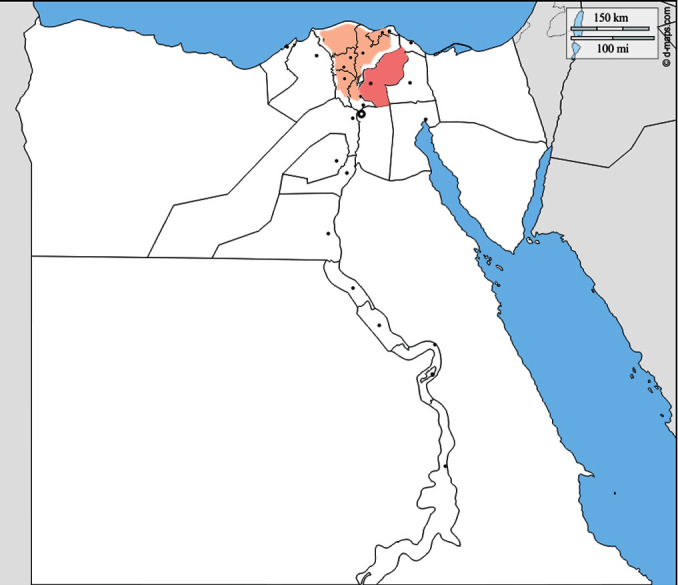
A map of Egypt depicting the study region. Sharkia governorate, which is located to the east of the Nile delta (orange color; https://d-maps.com/carte.php?num_car=4618&lang=en).

This study included 400 ready-to-eat vegetable samples comprising six types of leafy vegetables, namely lettuce (*Lactuca sativa*), radish (*Raphanus sativus*), coriander (*Coriandrum sativum*), parsley (*Petroselinum crispum*), dill (*Anethum graveolens*), and watercress (*Nasturtium officinale*), and four types of smooth surface vegetables, namely tomatoes (*Solanum lycopersicum*), green pepper (*Capsicum* species), cucumbers (*Cucumis sativus*), and carrots (*Daucus carota*). This study also investigated 180 samples of animal food: green fodder Alfalfa (*Medicago sativa*). These vegetables were chosen because of their widespread appeal among the population and large yearly raw consumption of different salad dishes. Fresh samples were obtained randomly from field areas in Sharkia governorate, Egypt, between January and December 2021. Sharkia governorate is Egypt's third most populated governorate that is located in the country's north, to the east of the Nile River Delta (Figure 1). This region, which is estimated to measure 4180 km^2^ (1610 square miles), is notable for its year-round cultivation of a diverse range of crops on thousands of acres of mud soils. Several irrigation canals run across the governorate, and many domestic animal species are reared indoors or in open agricultural areas. Summer and winter average temperatures are expected to be 27° and 18°, respectively. Winter rains are heavier in the north and west of the governorate, whereas the south and west receive the least. The collection of vegetables was done between the hours of 7 a.m. and 11 a.m. Weekly samples were taken, and all samples that were wilting, moldy, or withered were rejected. Sound fresh samples were placed separately into labeled sterile plastic bags without preservation and then transported to the closest laboratory as fast as possible. Two hundred grams of the edible section of each vegetable sample was prepared individually according to the routine household procedure.

#### 2.3.2. Soil Samples

Simple random method was used to gather soil samples. The collecting period coincided with the collection of vegetables. The number of samples collected from each field is determined by the vegetable growing area. In each collection, approximately 50 g was collected from a 5 cm ground depth. A total of 400 soil samples were obtained at ten different points per 100 m^2^. Soils were collected near vegetable plots and the vicinity of irrigation canals. No preservatives were added to the soil samples. Each soil sample was put in a plastic bag, tagged, and sealed before being sent to the laboratory. These locations were also chosen since they are bulk vegetable-producing sites. To enable weekly sample collection and transportation, site locations were chosen based on their closeness to the testing laboratory. All of the sites were in rural settings with open cultivation fields.

### 2.4. Techniques of Sample Preparation

#### 2.4.1. Vegetable Samples

Each 200 g of individual vegetable was soaked for fifteen minutes in 1 L of physiological saline, and then vigorously shaken for 5 minutes with the help of a mechanical shaker. After removing the vegetable sample, the washing water was filtered through a sterile 32–36 m hole-size sieve to eliminate undesired debris while allowing different sizes of parasite forms to pass [17]. The residual wash solution was allowed to settle for 8 hours. The filtrate was then emptied into clean centrifuge tubes and centrifuged for 10 minutes at 2000 rpm (447*g*). The supernatant was removed, and the sediment yielded parasitological examination to find various parasite eggs, larvae, cysts, and oocysts.

#### 2.4.2. Soil Samples

Because samples were damp, all samples were air-dried at room temperature for around 24 hours on a tray before being sifted using a 150-*μ*m filter to eliminate big particles.

### 2.5. Parasitological Examination and Estimation of Parasitic Eggs/Larvae/Oocysts

#### 2.5.1. Vegetable Sample Processing

Formal ether sedimentation concentration technique was utilized for the sediments, and stained and unstained smears were examined for parasite detection [29, 30]. For the unstained smear, a drop of the sediment was transferred on a newly cleaned slide, and a cover slip was delicately placed to minimize air bubbles and flooding. After that, the preparation was examined under a light microscope with 10×, 40×, and 100× objectives. For stained smears, a drop of the sediment was mixed with a drop of Lugol's iodine solution and inspected microscopically as in a simple smear. To identify cryptosporidium oocysts, modified Ziehl–Neelsen staining was used. It is a stain used to identify acid-fast organisms as Cryptosporidium spp. that stain red, whereas the background of debris stains blue [29]. The procedure was repeated until the mixture in each test tube was depleted.

#### 2.5.2. Soil Sample Processing

Modified flotation technique was used for soil samples [31, 32]. Briefly, 5 g of sieved soil were deposited in a test tube and rinsed with 8 ml distilled water by vortex mixer. After that, the suspension was centrifuged for 10 minutes at 1800 rpm. After decanting the supernatant, 8 ml of sucrose solution (specific gravity 1.2) was added to the sediment in the tube and vigorously mixed with a vortex mixer. The tube was centrifuged at 1800 rpm for 10 minutes. After centrifugation, a 10-ml syringe was used to progressively inject sucrose solution to the brim of the tube until an upper meniscus was formed. To collect the uppermost portion of the sucrose suspension, a cover slip was carefully put on the meniscus, and the prepared slides were checked for parasites using different stains and a light microscope at magnifications of 100×, 400×, and 1000×.

Preparation of all samples and identification of parasites were carried out in the Medical Parasitology Department Laboratory, Faculty of Medicine; Zoology Department Laboratory, Faculty of Science; and the Central Laboratory of Soil, Nutrition, and Fodder, Zagazig University, Egypt, using a binocular optical microscope in which the size of the parasitic structures was measured with the aid of an eyepiece and a micrometric ruler and compared with imaging data previously documented [29]. When parasites cannot be discovered in a sample, it is considered negative.

### 2.6. Data Analysis

Statistical analysis was performed with the SPSS software version 22 (IBM, Chicago, IL, USA). Data were presented in numbers and percentages. *P*-values equal to or less than 0.05 were considered to be statistically significant. The difference in parasitic contamination among the different categories was compared using the chi-square test.

## 3. Results

The occurrence of parasitic infection in soil nearby irrigation canals dispersed throughout the research region is shown in Table 1. More than half of the collected samples (60.7%) tested significantly positive for parasite contamination. The largest proportion of pollution was found in areas near the El-Senety canal (80%), whereas soil samples near the Moias canal area had significantly the lowest (40%). Nearly 40.8% of positive samples had one parasite species, whereas 37.9% and 11.5% of positive samples contained two and three parasitic species, respectively. These results were significant. From all positive samples, the probability of isolating more than three species did not surpass 10% (9.9%).

The detected parasitic contaminants were mainly *Escherichia coli* followed by *Cryptosporidium*, *Entamoeba histolytica/Entamoeba dispar* then *Ascaris* with significant presence. *Toxocara* spp. eggs, *Trichuris*, and *Fasciola* spp. eggs were insignificantly detected, and these findings incriminated both man and animal in soil contamination (Table 2).

Table 3 shows the number and type of freshly eaten vegetable samples that were thoroughly inspected, as well as the proportion of contaminated samples. When the 400 vegetable samples were tested for parasitic contamination, 249 (62.25%) of them significantly tested positive. About 80% of the parsley samples proved positive for parasites among all plants with uneven surfaces. Dill samples had the lowest proportion of parasitic contamination (41.7%). Green pepper had the highest level of contamination (67.5%) among vegetables with smooth surfaces, whereas cucumber samples had the lowest (50%). In this investigation, 65.1% of the vegetable samples proved positive for one parasite species. Similarly, 25.7% and 9.2% of positive samples tested positive for two and three parasite species, respectively, but a significant finding was recorded with the three parasitic species' contamination.

**Figure 2:**
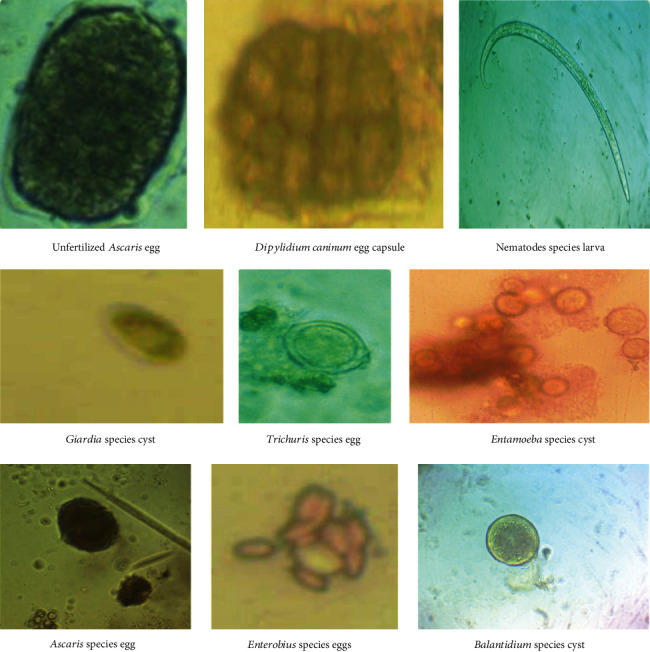
A plate showing the eggs/larvae/cysts of the identified parasites in fresh green vegetables samples.

Several parasites species (helminths and protozoa) were isolated from the studied vegetable samples in this investigation with significant results (Table 4 and Figure 2). Most positive samples contained *Ascaris* species eggs, *Trichuris* species eggs, and *Giardia* species cysts, which were predominantly isolated from vegetables with uneven surfaces. The parasite *Blastocystis hominis* was the least common in positive vegetable samples. The examined vegetables showed negative results for some parasites eggs, such as *Ancylostoma* spp. ova, despite the high prevalence of other soil-transmitted helminths, such as *Ascaris* and *Trichuris.*

Table 5 indicates the number and percentage of contaminated fresh green vegetable samples with parasite species discovered during different seasons. Throughout the year, we were able to isolate parasite forms from the investigated vegetable sample. Spring had the highest percentage of parasitic infection in vegetable samples (29.3%), followed by summer (27.7%) and autumn (24.5%). The winter season has the lowest prevalence rate (20.1%). Only the results of autumn were significant.

**Figure 3:**
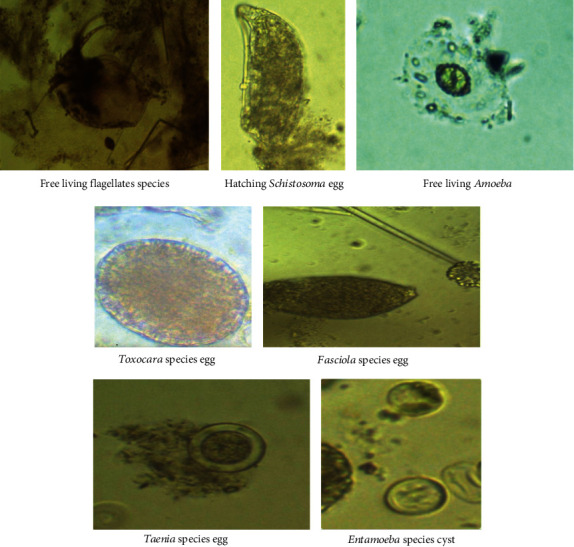
A plate showing the eggs/larvae/cysts of the identified parasites in green fodder (*Alpha alpha*).

Table 6 shows a significant number and percentage of positive green fodder samples with parasite species found (Figure 3). It is worth noting that no green fodder is seeded throughout the summer. As a result, no results were recorded during this season. Many parasite species have been discovered to contaminate the more prevalent animal diet in Egypt, with the most frequent parasite reported being Nematode spp. larvae. Different intestinal helminthic eggs have also been discovered, indicating fecal matter contamination.

## 4. Discussion

Human health is primarily determined by the nutritional quality of the food they consume daily. The governmental food and health sectors are responsible for the hygienic and sanitary conditions of the food-producing chain, from the field to the consumer, which necessitates constant tracking of any diseases. Among food-borne pathogens, food-borne parasites are of special worldwide significance [33]. Sharkia is a big agricultural governorate in Egypt, that cultivates and its population consumes a variety of green vegetables. The present study attempted to explore the probability of parasite contamination of the farming mud soil of this area and some green vegetables that are regularly ingested raw and utilized daily in salad dishes. In the current study, concentration, staining, and light microscopy examination of vegetables and environmental materials comprising soil for parasites revealed varying contamination rates with helminths and protozoa of different species.

Soil is a key source of people and animal infection with different parasites of public health significance. The current investigation's findings, which showed the existence of parasitic life forms in all of the places where soil samples were collected, substantiate this. Over half of the soil samples collected (400) were significantly positive for at least one parasite type. This finding is backed by the fact that mud soil allows most parasites to survive for a long time because it has enough moisture and little sunlight, and the tightly adhering particles prevent parasitic larvae from moving to deeper levels of soil [32]. Therefore, we were able to detect parasitic structures from the majority of samples obtained. Similar studies on soil were undertaken in other countries [17, 22, 34, 35] and revealed a large number of parasitic larvae, oocysts, and different eggs contaminating any type of soil, including farming lands. It is difficult to separate parasitic structures that may be trapped in the particle material of soil samples. An intriguing issue in this study is that we cannot rule out the presence of parasites in negative soil samples obtained in this study since samples with a high concentration of particles (including soil particles) might readily trap or get attached to the parasitic structures, resulting in reduced recovery and viewing [17]. This also might be attributable to the random sampling technique of soil used in this study, since some samples may have been taken distant from hot spots of fecal material, which represent the starting point of diffusion [32]. Our study results add to the evidence that soil might serve as a source of contamination for human and animal parasitic infection in the region.

The most important factor that may contribute to the contamination of soil in farming areas is irrigation water. This is of great concern where contamination of irrigation water with fecal material is a likely possibility to occur or the use of treated wastewater is a must due to scarcity in freshwater supply. The use of treated sewage water could be a major source of contamination of the soil with parasitic eggs as well as the growing vegetables [26]. In addition, our observation of the irresponsible behavior of some individuals who insist to dump human and animal farming waste along the irrigation streams could aid in soil contamination. This factor is of great importance in our studied area where the main source of irrigation water is surface water bodies, which can bring the fecal matter of humans and livestock to downstream vegetable farmlands. Most of the buried parasites can remain infective for a long time in soil due to their resistant wall and can be distributed to vegetable farmlands with future liability to infect human hosts [36]. As in other Sub-Saharan African cities, the lack of adequate sanitation and drainage infrastructure [37] causes contamination of water bodies used for irrigation, which in turn contaminates the soil that becomes a vehicle for human infection [38]. Many studies document soil contamination with different parasite structures either due to accidental and unintended exposure to infected water sources [17] or due to intended human practice to infect soil with fecal material as a part of organic farming practices [22, 34] with STHs (*Ascaris* and *Trichuris*) alongside with *Cryptosporidium* spp., *Toxocara* spp., and *Entamoeba* spp. being the most prevalent parasites in soil samples collected. In addition, the presence of animals during farming, as well as the use of their manure as fertilizer, can be a potential cause of soil contamination with parasites. Due to the large livestock development in the area of the study, the use of manure of animal wastes is a tradition in fertilizing the farmlands. The quantity of farm animals is directly linked to the rate of soil contamination with parasite life forms [34]. It is worth mentioning that the soil depth from which the samples are obtained greatly affects the rate of recovery of parasitic structures from soil samples. In the present study as well as in other works, most of the samples were obtained from the top 0–5 cm layer of the soil, which may result in great recovery of parasites as it gives the optimum environment for most of the parasites especially eggs of soil-transmitted helminths. Nevertheless, depths up to 10 cm have no substantial influence on the rate of parasitic structure recovery from soil [22]. In addition, soil type could greatly influence the recovery of parasitic life forms, particularly helminthic eggs from the soil. It is thought that the homogenous loose nature of the large particles of sandy soils is reported to result in higher recoveries of parasites than other soil types [17].

Fresh green vegetables, particularly those consumed raw in salad dishes or barely cooked are a major source of intestinal parasite transmission and have been connected to food-borne parasitic infections in many communities throughout the world [10, 39]. Contamination most likely occurs before harvest while they are in fields, either by unregulated use of contaminated raw human or animal manure, sewage sludge, infected irrigation water, or directly through unsanitary hygienic practices of humans, such as open defecation in farmlands. All of these probable contamination occurrences are feasible vectors of parasite transmission and compatible with the premise that the contamination level of soil and irrigation water is high in developing countries, which has an impact on the safety of growing plants [40]. Because these parasites employ a broad variety of transmission pathways, the life cycle phases found in food and used as detection targets vary substantially. In the 400 samples of green vegetables examined, nine parasites from the metazoa and protozoa groups were identified. Metazoa (helminths) included *Ascaris* spp., Nematode spp. larvae, *Trichuris* spp., and *D. caninum*, whereas protozoa included *Blastocystis* spp., *Balantidium* spp., *Entamoeba* spp., *Giardia* spp., and *Cryptosporidium* spp. These parasite entities are emphasized as key causal factors of infection conveyed by vegetable-derived meals [28].

In this study, we were able to detect parasitic species from most of the leafy greens compared with other vegetables investigated. It is stated that leafy vegetables like parsley and coriander are typically cultivated and harvested close to the soil, exposing them to polluted soil, contaminated irrigation water, or untreated manure. Moreover, these plants have irregular surfaces and dense foliage that guarantee a heavy parasitic load in such plants in contrast to vining crops like tomatoes, cucumber, and green pepper that are usually grown and collected far from the ground and have a smooth surface that does not support the attachment of parasites and easily cleaned of residual soil [41]. This premise could work in many research papers where they were able to isolate more parasites from leafy vegetables than other vegetable staffs [17, 18, 42]. We further credit this finding to the procedure used to recover the parasites, as we soaked vegetables in saline, subjected them to vibration, and separated them into leaves, which assisted in the isolation of many parasites in the washing solution. This does not happen in real life since most customers disregard soaking vegetables in a bowl of tap water and cleaning them numerous times with their hands or by strong agitation and are satisfied with a single exterior wash. This finding emphasizes the necessity of washing vegetables, which reduces the rate of parasite infection [43]. In this study, 66.7% of carrot samples reported positive for parasites as these root vegetables are in close association with contaminated soil and are more exposed to infected irrigation water [17].

Significant differences in the occurrence of these parasites in vegetables have been documented globally. These disparities might be related to variances in ambient and climatic circumstances, hygienic settings, laboratory procedures used, and the types of vegetables collected, all of which have a role in parasitic disease transmission. Over the past two decades, many research studies published in the developing [15, 16, 44, 45] and developed [46] countries reported the presence of these parasites in various staffs of green vegetables at various points along the production chain, from the fields to the consumer's table suggesting that green vegetables might serve as important vehicles for parasites transmitted by food.

In this study, the existence of human and animal intestinal parasites demonstrates that different contamination sources greatly impact the continual contamination of vegetables grown in open fields. Inadequate human sanitation practices, such as wastewater and sewage treatment, as well as contamination of agricultural soil, owing to the use of polluted irrigation water sources with fecal materials are heavily implicated in this situation. It appears that these key variables had a long-term influence on vegetable safety since the same parasites were identified in the research location [47] and other geographical regions of Egypt, over years [44, 48]. Despite, the contamination of vegetables can occur in a variety of ways, it is most commonly related to irrigation water. The irrigation of vegetables using sewage-contaminated water is a prevalent practice in impoverished nations. This argument (irrigation with contaminated water) is emphasized by the fact that even vegetables cultivated in secure and closed-off regions are susceptible to parasitic contamination via unfiltered or wastewater used for irrigation [49]. The movement of intestinal parasites from their primary hosts to different vegetables emphasizes the need of implementing in-field hygiene measures to disrupt the parasite life cycle and reduce the possibility of fecal–oral channel transmission [7]. A larger-scale assessment of the green vegetable production process and a review of the community's biosecurity concepts and practices may restrict the potential for pathogen contamination on farms, markets, and even at home, ensuring safe food for consumption [3].

Although a definitive comparison between the prevalence of parasites in investigated vegetables and soil was not achieved in our study due to difficulties in transporting the same vegetable of the same soil sample, the hypothesis that any parasite contaminating soil could be transferred to the cultivated vegetable crops, which are then consumed remains possible [50]. Interestingly, a study in the Philippines detected the parasitic contamination of both vegetables and soil samples obtained from the same study sites [51]. It is crucial to remember that food-borne parasites have multiple transmission channels, therefore, the forms of their life cycle discovered in different food staffs and used as detection targets vary greatly. Furthermore, because of minimal reproduction chances outside of hosts (animals and people) and the small infectious dosages needed for establishing infection, detecting tiny quantities of parasites is critical. The majority of detection techniques in environmental samples do not show the viability or infectivity of parasites in humans. Generally, the minimum number of infective oocysts/eggs required for infection establishment is not precisely determined for most parasites, but it is estimated to be low (10), hence ensuring the accuracy of a negative diagnosis is critical.

In this study, eggs of *Ascaris* species were the most prevalent species in the examined vegetables. They are among the most common parasites that contaminate vegetables and other foods [18] and have priority management because they infect numerous populations globally via the oral route of transmission [33]. These eggs have been identified in environmental samples contaminated with fecal content, such as water; however, the ova settle rapidly out of the water column and inhabit the soil with possible transmission to growing plants and individuals who are in contact with infected soil [38]. As a result, it is expected that the high incidence of *Ascaris* eggs in plants and soil is heavily impacted by human infection rates. This high prevalence may be linked to the adult female's great fecundity since it lays around 200,000 eggs every day to maximize its chances of transmission to subsequent hosts. Furthermore, the thick-shelled nature of their eggs makes them more resistant to many unfavorable situations, such as chemical exposure and dehydration, helping them to tolerate severe environmental conditions and survive longer in soil habitats required for growth [22]. We believe that the temperature of the research region influences the growth of this parasite in soil, providing it more opportunities to live and contaminate the environment, including the growing vegetables. These eggs have been shown to maturate optimally around 32°C and can survive in ambient samples for more than ten months in tropical climates [52]. This extended living period is sufficient to risk individuals who handle or consume fresh produce products, particularly when survival time exceeds the development cycle, as is frequently the case with the investigated vegetables [53]. Because of the sticky nature due to mamillated eggshell surface, *Ascaris* egg, it is the most commonly identified parasite in vegetables throughout the production chain, not only during farming [15]. The second most prevalent parasite detected in our vegetable samples is the *Trichuris* species. This is due to the high fertility rate of single female helminthic worms, which allows their eggs to infect a wide area of soil. Furthermore, their thick-shelled eggs can withstand severe temperature and moisture fluctuations [54]. It is noteworthy that the detection of *D. caninum* eggs in the vegetable samples is an important indication of zoonotic infections from infected animals, particularly dogs and cats [17]. It is important to mention that non-infective life forms, such as the un-embryonated ova of *Ascaris* and *Trichuris* species, have been identified in this study, which might develop into infective stages over time and poses major health hazards. Although helminth egg load concentrations were not detected in this investigation, it is reported that just one embryonated helminthic egg may be sufficient to establish infection [55].

*Giardia* spp. cysts were the most prevalent protozoa recovered from the examined vegetables (41.8%), followed by *Cryptosporidium* spp. oocyst (26.5%) and *E. histolytica/E. dispar* cysts (22.1%), respectively. These parasites have been identified in a variety of foods and have been linked to green vegetables in several countries throughout the world [46, 56]. Water and food-borne transmission are significant factors in these parasites' prevalence in human communities. These pathogenic protozoa may have infected green vegetables through the soil since *E. histolytica* may survive in the environment for eight days at temperatures ranging from 28°C to 34°C, and *Giardia lamblia* can survive in the water for up to one month at 21°C [33]. Many research papers show the preponderance of *Giardia* among protozoa parasites tested on fresh food at the farm, which is comparable with our findings [10, 57]. The low infective dose of these parasites and their ability to replicate inside human host has been linked to several food-borne outbreaks, particularly in the developed countries [58], prompting international food safety agencies to develop a standard International Organization for Standardization (ISO) protocol for identifying them in leafy greens and berry fruits [59]. The altered epidemiological situation caused by the interaction of agriculture border expansion, natural ecosystem changes, and the introduction of animal species for food production all had a significant impact on the prevalence of these protozoa in humans [24, 60]. *B. coli* and *Blastocysts* species were the least common parasites detected in this investigation, accounting for 18.2% and 11.2% of positive samples, respectively. Food-borne transmission of *B. coli* is of no significant importance in the developed countries, as this parasite has rarely been implicated in food-borne outbreaks; however, it has been frequently reported in surveys of parasites in fresh vegetables eaten raw in developing countries [61].

The rate of parasite infection in vegetable samples was assessed in this study in response to seasonal variation. Spring had the largest percentage of positive samples (29.3%), followed by summer (27.7%) and autumn (24.5%). Winter had the lowest rate of positive samples (20.1%). Significant variations in the prevalence of these parasites have been observed in vegetables due to temperature and moisture fluctuations all over the year. Our findings were consistent with previous studies in Egypt [44, 62], and other countries [46, 63–65] that report a high number of contaminated samples in dry seasons (spring and summer) in comparison with wet seasons (winter and autumn). Another study in Hanoi, Vietnam, showed that the number of eggs recovered from vegetables was higher in the dry season (78%) than in the rainy season (22%) of a total number of eggs recovered [66]. This might be owing to the increased presence of parasites, notably STH, in soils during dry seasons; furthermore, a lack of rain reduces the wash of eggs away from the soil and the vegetables and results in the need for an additional irrigation cycle [32]. However, a study in Turkey, found that rain had a favorable effect on the parasite burden of vegetables, contradicting this hypothesis [42]. According to our observations, it is obvious that the light rain in autumn in our location has a potentially positive effect on the parasite burden of vegetables detected in this study.

Although there are many research papers in the literature that discuss the rate of parasite contamination of soil and vegetables along the production-consuming chain, inter-study comparisons are impossible because the majority of these publications lack proper validation and unified parasitological sample processing, particularly for protozoa. The methodologies employed in studies for the isolation of parasites in environmental samples range from traditional microscopy-based approaches used to observe the parasite to advanced DNA detection methods Polymerase Chain Reaction, which may result in a variation in the rate of parasites isolated [3]. Using molecular methods in studies performance helps in genus differentiation of different parasites, such as nematodes larvae, they have many sources like plant *Nematoda*, soil free-living nematodes in addition to parasitic nematodes.

Other important considerations include the kind and size of vegetable samples, the quality of water used for irrigation and washing, the proximity of livestock, and the availability of organic fertilizers. Even producers' and consumers' knowledge of the risk of parasite transmission through vegetables, as well as the population's cleanliness practices, might impact the detection of parasites in green vegetables [67]. The main limitation of our study, as well as the majority of papers discussing the prevalence of parasites in environmental samples, such as soil and vegetables, is the lack of molecular characterization of the identified parasites. This identification is crucial for source attribution and comparison with infections in humans, which is critical in outbreak investigations. In the present work, neither viability nor infectivity is displayed, which is a crucial parameter that largely determines the rate of infectivity in human populations and the validity of the sanitation measures [10]. It is worth noting that several green fodder samples were taken and tested for parasite infestation during our study. Many parasites have been documented to infect the most common animal food in Egypt, such as *Nematode* spp. larvae, being the most often reported contaminant of the collected samples. Various intestinal helminthic eggs were also detected, indicating fecal matter contamination. These findings indicate the potential infectivity of this plant to both animal hosts and humans who handle or come into contact with it during the transportation or animal feeding process.

## 5. Conclusions and Recommendations

This study backs up earlier findings of parasitic contamination of green vegetables, fodder, and soil in Egypt, emphasizing the importance of frequent inspection of irrigation water control and monitoring the efficacy of wastewater treatment procedures. The detection of metazoa and protozoa parasites species in this study underlines the necessity for fresh product producers to be more health educated to reduce the dangers of fresh food contamination with human and animal feces. Because fresh produce is frequently consumed without further thermal processing, health officials must encourage consumers to take appropriate sanitary precautions while handling or eating vegetables to decrease the possibility of ingesting parasites' infective stages. More epidemiological and surveillance research studies on food-borne parasites are to be encouraged through total collaboration between academic research and the food industry to standardize efficient experimental approaches needed to isolate parasites from different environmental samples. Any progression in genetic identification methodologies of the analytical protocols of environmental samples would help not only to assess the level of environmental contamination, but also to identify the parasite genus and species, and determine the viability and subsequent potential infectivity of the investigated parasites. All of these approaches would increase understanding of the food-borne transmission route, risk assessments, and the identification and verification of crucial control points.

## Figures and Tables

**Table 1:** The number and percentage of positive soil samples contaminated with parasite species.

Location	Number of examined samples	Number of contaminated samples	Percentage of contaminated samples	Polyparasitism parasite species detected *n* (%)			
				1 species	2 species	3 species	>3 species
El-Ganabia canal area	80	50	62.5	6 (12.0)∗	30 (60.0)	10 (20.0)	4 (8.0)
El-Mahmodyia canal area	70	53	75.5	13 (24.5)	25 (47.2)	10 (18.9)	5 (9.4)
El-Senety canal area	50	40	80.0	33 (82.5)	4 (10.0)∗	3 (7.5)	0 (0.0)
Moias canal area	100	40	40.0∗	17 (42.5)	13 (32.5)	5 (12.5)	5 (12.5)
Saft Zerek canal area	100	60	60.0	30 (50.0)	20 (33.3)	0 (0.0)	10 (16.7)
Total	400	243	60.7	99 (40.8)	92 (37.9)	28 (11.5)	24 (9.9)
Test			32.53	53.9	26.58	14.82	8.01
*P*-value			0.001∗	0.001∗	0.001∗	0.005∗	0.09

∗Significant *P*-value <0.05.

**Table 2:** The parasites contaminating the soil in the studied localities.

Detected parasite	El-Ganabia	El-Mahmodyia	El-Senety	Sherwida	Saft canal	Total	Chi-square	*P*-value
*Fasciola* spp.eggs	2	4	4	1	6	17	3.2727	0.351
*Ascaris* eggs	5	8	12	1	3	29	13.3333	0.003∗
*Toxocara* spp. eggs	3	3	8	2	4	20	7.3333	0.061
*Trichuris* eggs	0	1	2	0	3	6	1.6593	0.646
*Cryptosporidium* spp. oocysts	8	12	10	2	13	45	9.3333	0.025∗
*E. histolytica/E. dispar* cyst	7	7	12	2	5	33	9.5238	0.023∗
*E. coli* cyst	14	16	12	2	20	64	14.0606	0.002∗
*Giardia* cyst	3	4	4	3	3	17	0.381	0.944
*Cyclospra* spp. oocyst	1	1	2	0	3	7	0.80	0.849
*Isospora* spp. oocyst	1	1	1	0	2	3	0.11	1.00
Total	44	57	67	13	62	243	0.02	0.05

∗Significant *P*-value <0.05.

**Table 3:** The number and type of totally examined freshly eaten vegetable samples as well as the proportion of contaminated samples.

Type of vegetables	Item	Number of examined samples	Number of contaminated samples	Percentage of contaminated samples	Polyparasitism parasite species detected *n* (%)		
					One species	Two species	Three species
Irregular surface	Coriander	50	31	62.0	22 (71.0)	7 (22.6)	2 (6.5)
	Dill	50	25	50.0	20 (80.0)	4 (16.0)	1 (4.0)
	Lettuce	40	27	67.5	17 (63.0)	7 (26.0)	3 (11.1)
	Radish	40	30	75.0	20 (66.7)	10 (33.3)	0 (0.0)
	Parsley	40	32	80.0	22 (68.8)	9 (28.1)	1 (3.1)
	Watercress	50	22	44.0	12 (54.5)	7 (31.9)	3 (13.6)
Regular surface	Carrot	30	20	66.7	9 (45.0)	5 (25.0)	6 (30.0)
	Cucumber	30	15	50.0	10 (66.7)	4 (26.7)	1 (6.7)
	Tomatoes	30	20	66.7	11 (55.0)	5 (25.0)	4 (20.0)
	Green pepper	40	27	67.5	19 (70.3)	6 (22.2)	2 (7.4)
Total		400	249	62.25	162 (65.1)	64 (25.7)	23 (9.2)
Test			26.86		9.06	3.02	19.48
*P*-value			0.0001∗		0.43NS	0.96NS	0.02∗

∗Significant *P*-value <0.05; NS = non-significant *P*-value >0.05.

**Table 4:** The percentage and type of parasitic species detected in the freshly eaten vegetables positive samples.

Parasite	Number of positive samples (total = 249)	Percentage of positive samples (%)	Coriander *n* = 31	Dill *n* = 25	Lettuce *n* = 27	Radish *n* = 30	Parsley *n* = 32	Watercress' *n* = 22	Carrot *n* = 20	Cucumber *n* = 15	Tomatoes *n* = 20	Green pepper *n* = 27	*P*-value
Helminths													
*Ascaris* spp. eggs	205	82.3	26	25	25	27	30	20	12	9	11	20	NS
*Trichuris* spp. egg	100	40.2	10	11	11	12	14	14	8	6	9	5	NS
Nematode spp. larvae	55	22.1	10	7	0	2	6	9	8	4	4	5	NS
*Dipylidium caninum* spp.	33	13.2	6	8	7	2	4	3	0	0	3	0	NS
Protozoa													
*Giardia* spp. cyst	104	41.8	13	12	15	7	10	10	14	9	6	8	NS
*Cryptosporidium* spp. oocyst	66	26.5	7	10	8	5	9	10	4	4	1	8	NS
*Entamoeba* spp. cyst	55	22.1	0	0	9	10	6	8	6	9	8	9	NS
*Balantidium coli* cyst	45	18.2	5	6	6	0	8	3	7	5	0	5	NS
*B. hominis* spp.	28	11.2	3	6	4	3	2	1	1	3	3	2	NS
*P*-value	<0.001∗∗												

∗∗Significant *P*-value <0.05; NS = non-significant *P*-value >0.05.

**Table 5:** The number and percentage of positive fresh green vegetables samples contaminated with parasites species detected during different seasons.

Vegetable	Total number of examined samples	Number of positive samples	Spring (total = 100)		Summer *n* = 100		Autumn *n* = 100		Winter *n* = 100		Test	*P*-value
			*N*	%	*N*	%	*N*	%	*N*	%		
Coriander	50	31	12	38.7	8	25.8	6	19.4	5	16.1	1.78	0.6 NS
Dill	50	25	10	40.0	6	24.0	4	16.0	5	20.0	1.87	0.6 NS
Lettuce	40	27	8	29.6	8	29.6	5	18.5	6	22.2	0.55	0.9 NS
Radish	40	30	10	33.3	8	26.7	7	23.3	5	16.7	0.41	0.9 NS
Parsley	40	32	9	28.1	10	31.2	4	12.5	9	28.1	3.56	0.3 NS
Watercress	50	22	7	31.8	6	27.2	7	31.8	2	9.1	2.06	0. 6 NS
Carrot	30	20	3	15.0	6	30.0	7	35.0	4	20.0	2.57	0.5 NS
Cucumber	30	20	5	33.3	2	13.3	8	53.3	5	33.3	5.06	0.2 NS
Tomatoes	30	20	3	15.0	6	30.0	7	35.0	4	20.0	2.57	0.5 NS
Green pepper	40	27	6	22.2	9	33.3	7	25.9	5	18.5	0.93	0.8 NS
Total	400	249	73	29.3	69	27.7	61	24.5	50	20.1		
Test			7.75		2.57		20.11		11.7	
*P*-value			0.5NS		0.9NS		0.01∗		0.2NS	

∗Significant *P*-value <0.05; NS = non-significant *P*-value >0.05.

**Table 6:** The number and percentage of positive green fodder samples with the most often parasite species detected.

	Number of examined samples	Number of contaminated samples	Percentage of contaminated samples	The most common parasites species detected		
				Winter	Spring	Autumn
El-Ganabia canal area	20	10	50.0	—	Free-living flagellate spp.	—
					Nematode spp. larvae	
El-Mahmodyia canal area	50	30	60.0	*Fasciola* spp. eggs	*Taenia* spp.	Free-living flagellates spp.
					Nematode spp. larvae	
El-Senety canal area	20	15	75.0	Granular form free-living *Amoeba* spp.	—	—
Moias canal area	40	19	47.5	*Toxocara* spp.	—	*Ascaris* spp. eggs
				*Entamoeba* spp. cyst		*Balantidium* spp. cyst
Saft Zerek canal area	50	35	70.0	*Schistosoma* spp. eggs	*Taenia* spp. eggs	Nematode spp. larvae
					Nematode spp. larvae	*Fasciola* spp. eggs
Total	180	109	60.6			
Chi-square test	7.41					
*P*-value	0.11NS∗∗					

∗∗NS = Non-significant *P* value >0.05.

## Data Availability

Data available on request through contacting the corresponding author Dr. Samah Yahia (Emails: parasitologistsamah@yahoo.com; SHYehya@medicine.zu.edu.eg).
